# A Feasibility Study of Expanded Home-Based Telerehabilitation After Stroke

**DOI:** 10.3389/fneur.2020.611453

**Published:** 2021-02-03

**Authors:** Steven C. Cramer, Lucy Dodakian, Vu Le, Alison McKenzie, Jill See, Renee Augsburger, Robert J. Zhou, Sophia M. Raefsky, Thalia Nguyen, Benjamin Vanderschelden, Gene Wong, Daniel Bandak, Laila Nazarzai, Amar Dhand, Walt Scacchi, Jutta Heckhausen

**Affiliations:** ^1^Department of Neurology, University of California, Los Angeles, Los Angeles, CA, United States; ^2^California Rehabilitation Institute, Los Angeles, CA, United States; ^3^Department of Neurology, University of California, Irvine, Irvine, CA, United States; ^4^Department of Physical Therapy, Chapman University, Orange, CA, United States; ^5^Department of Neurology, Brigham and Women's Hospital, Boston, MA, United States; ^6^Institute for Software Research, University of California, Irvine, Irvine, CA, United States; ^7^Department of Psychological Science, University of California, Irvine, Irvine, CA, United States

**Keywords:** stroke, telehealth, recovery, rehabilitation, holistic

## Abstract

**Introduction:** High doses of activity-based rehabilitation therapy improve outcomes after stroke, but many patients do not receive this for various reasons such as poor access, transportation difficulties, and low compliance. Home-based telerehabilitation (TR) can address these issues. The current study evaluated the feasibility of an expanded TR program.

**Methods:** Under the supervision of a licensed therapist, adults with stroke and limb weakness received home-based TR (1 h/day, 6 days/week) delivered using games and exercises. New features examined include extending therapy to 12 weeks duration, treating both arm and leg motor deficits, patient assessments performed with no therapist supervision, adding sensors to real objects, ingesting a daily experimental (placebo) pill, and generating automated actionable reports.

**Results:** Enrollees (*n* = 13) were median age 61 (IQR 52–65.5), and 129 (52–486) days post-stroke. Patients initiated therapy on 79.9% of assigned days and completed therapy on 65.7% of days; median therapy dose was 50.4 (33.3–56.7) h. Non-compliance doubled during weeks 7–12. Modified Rankin scores improved in 6/13 patients, 3 of whom were >3 months post-stroke. Fugl-Meyer motor scores increased by 6 (2.5–12.5) points in the arm and 1 (−0.5 to 5) point in the leg. Assessments spanning numerous dimensions of stroke outcomes were successfully implemented; some, including a weekly measure that documented a decline in fatigue (*p* = 0.004), were successfully scored without therapist supervision. Using data from an attached sensor, real objects could be used to drive game play. The experimental pill was taken on 90.9% of therapy days. Automatic actionable reports reliably notified study personnel when critical values were reached.

**Conclusions:** Several new features performed well, and useful insights were obtained for those that did not. A home-based telehealth system supports a holistic approach to rehabilitation care, including intensive rehabilitation therapy, secondary stroke prevention, screening for complications of stroke, and daily ingestion of a pill. This feasibility study informs future efforts to expand stroke TR.

**Clinical Trial Registration:** Clinicaltrials.gov, # NCT03460587.

## Introduction

Stroke is perennially among the leading causes of human disability (1, 2) and the leading neurological cause of lost disability-adjusted life years (3). The number of affected people has doubled over the past two decades (4), partly because of the aging population (5) and partly because advances in stroke medicine have increased the fraction of patients surviving acute stroke (6). Motor deficits, present in >80% of patients with stroke acutely, are a major contributor to this disability. Few patients recover completely, with 55–75% having enduring motor deficits (7, 8). At 6 months post-stroke, 65% of patients are unable to incorporate the paretic hand effectively into daily activities (9). Persistent arm impairment is linked with greater activity limitations, higher participation restrictions, poorer quality of life, and reduced well-being (10–12).

There is strong evidence that higher doses of rehabilitation therapy are associated with greater behavioral gains, especially for paretic arm function after stroke (13–18), even with variability in treatment content and definition of dose (19). This remains true when higher therapy doses are delivered in the home (14, 20). However, patients generally do not receive high doses of rehabilitation therapies, due to cost, traveling difficulties, and regional shortages of rehabilitation providers–factors that are exacerbated in the COVID-19 era. Quality of rehabilitation therapy is also important and can increase the extent to which clinical neuroplasticity is harnessed (21): effects are higher when therapy is challenging, motivating, and engaging (22–25).

Telehealth might be able to help by increasing access to high quality therapy (26). Telerehabilitation (TR) has been defined as the delivery of rehabilitation services via communication technologies and encompasses a range of rehabilitation and habilitation services that include evaluation, assessment, monitoring, prevention, intervention, supervision, education, consultation and coaching (27). This is similar to the holistic framework outlined by Demiris et al. (28), who suggested that home-based post-stroke TR should include support that spans an array of medical, mental health, and social services. Compared to traditional in-clinic therapy, TR uses the same principles of individualized care by a licensed therapist. This telehealth approach provides enhanced options compared to delivery of rehabilitation services using a brick-and-mortar approach (29–33), potentially decreasing transportation needs for patients with functional limitations, boosting physical activity, and expanding access to care.

Telehealth can also help by increasing motivation and compliance. The technological underpinnings of TR can facilitate a personalized approach to upper extremity (UE) motor rehabilitation (34). Telehealth can deliver therapy in the form of games, an approach known to promote patient participation in health care (35–39). Games motivate patients to engage in enjoyable play behavior that involves therapeutically relevant movements (40, 41), which is important because patient compliance with stroke rehabilitation is often limited (42–44).

The overall experience with motor TR after stroke is mixed. While one review found that all 18 studies of post-stroke motor TR improved disability (32), a recent meta-analysis concluded that drawing general conclusions about the effects of stroke TR is difficult, as interventions and comparators varied greatly across studies (45). We have completed three trials of TR targeting arm motor deficits after stroke. The first was a pilot study (46) that provided 12 patients with chronic stroke with 4 weeks of home-based, therapist-supervised TR. Findings included that patients were highly compliant (97.9% of assigned days), videoconferences supported regular communication between the patient at home and therapists in the clinic, arm motor status improved significantly based on the UE Fugl-Meyer (UE-FM) motor scale, and no computer skills were needed, as computer literacy was not related to usage or treatment gains. With 60 min/day of TR, patients averaged 879 arm repetitions/day. A second study found that eight sessions of visuomotor training in the home improved visuomotor tracking by the UE (47).

More recently we led an 11-site, randomized, assessor-blind trial of TR (48). A total of 124 patients with stroke were randomized to receive 36 sessions of 70-min duration, either in-clinic or in the home via TR. In the 62 patients randomized to TR, UE-FM scores increased by 7.9 ± 6.7 points, and TR was found to be non-inferior to in-clinic therapy. Motor gains remained significant when patients enrolled >90 days post-stroke were examined separately. Gains were also significant when examining change in Box & Blocks score, a measure of arm function (activities limitation). In a separate manuscript under review, we found that 39.5% of patients randomized to TR and enrolled >90 days post-stroke showed reduced global disability (improved mRS score); in contrast, natural history data indicate that mRS scores generally plateau by day 90 (49, 50), suggesting that TR benefits might generalize to improved global functional outcomes.

The purpose of the current study was to evaluate the feasibility of several expansions to our prior TR program, in two main ways, treatment and assessment. Treatment topics were extension of daily TR from 6 to 12 weeks; incorporation of therapy targeting the lower extremity (LE), in addition to UE therapy; incorporation of augmented reality (AR) into this TR system; introduction of games that use a real object to train instrumental activities of daily living (iADLs); and addition of a daily study pill to be taken at the start of TR, a feature that might improve secondary stroke prevention and also might facilitate clinical trials of restorative therapies that are administered in pill form. Assessment topics included addition of tests performed by the patient using the TR system independently, with no therapist present; validation of telehealth screening for depression and aphasia; and generation of actionable email reports to clinicians whenever a critical finding occurred. The feasibility of each of these expansions was examined.

## Methods

### Study Overview

In this prospective, single-group, therapeutic feasibility trial, patients underwent live assessment at the UC Irvine clinic twice at baseline, after which a telehealth system was delivered to the patient's home. Patients then received 12 weeks of TR therapy, 6 days/week, with a live clinic assessment at the end of week 6 and week 12. Patients were free to call the lab with questions. This study was approved by the UC Irvine IRB, and was registered as clinicaltrials.gov ID # NCT03460587.

### Participants

Patients were recruited from the community through local advertisements. In sum, enrollees were adults with arm paresis due to stroke and no limiting cognitive deficits. Full entry criteria appear in Table 1. Patients signed informed consent (no surrogate consent) and were evaluated for eligibility at the first two visits.

**Table 1:** Entry criteria.

**Inclusion criteria**
1. Age ≥18 years at the time of randomization
2. Stroke that is radiologically verified, with any time of stroke onset prior to randomization
3. Upper extremity motor Fugl Meyer (UE-FM) score of 28–66 out of 66; to insure some deficit is present, if UE-FM > 59, must also have Box & Blocks (B&B) score on affected side >25% lower than on non-affected side
4. Box & Block Test score with affected arm is at least 3 blocks in 60 s at the first visit
5. Informed consent and behavioral contract signed by the subject
**Exclusion criteria**
1. A major, active, coexistent neurological or psychiatric disease, including alcoholism or dementia
2. A diagnosis (apart from the index stroke) that substantially affects paretic arm function
3. A major medical disorder that substantially reduces the likelihood that a subject will be able to comply with all study procedures
4. Severe depression, defined as Geriatric Depression Scale Score > 11 out of 15
5. Significant cognitive impairment, defined as Montreal Cognitive Assessment score < 22; this can be waived at the discretion of the study PI, e.g., for aphasia
6. Deficits in communication that interfere with reasonable study participation
7. Lacking visual acuity, with or without corrective lens, of 20/40 or better in at least one eye
8. Life expectancy < 6 months
9. Receipt of Botox to arms, legs, or trunk in the preceding 6 months, or expectation that Botox will be administered to the arm, leg, or trunk prior to completion of participation in this study
10. Unable to successfully perform all 3 of the rehabilitation exercise test examples
11. Unable or unwilling to perform study procedures/therapy, or expectation of non-compliance with study procedures/therapy, or expectation that subject will be unable to participate in study visits
12. Concurrent enrollment in another investigational study
13. Subject does not speak sufficient English to comply with study procedures
14. Expectation that subject will not have a single domicile address during the 12 weeks of therapy, within 75 miles of the central study site

### Study Intervention

After all eligibility criteria were confirmed, the patient signed a behavioral contract (51) that listed a personal treatment goal and the time when therapy would begin each day. An initial treatment plan was created by a licensed occupational therapist (OT) or physical therapist (PT), standardized by use of an algorithm that uses the 33 UE-FM sub-scores to identify the three greatest UE impairments. The algorithm suggests games and exercises that are matched to these three impairments and so calibrates initial TR games and exercises to each patient's impairment level.

Patients were provided 72 treatment sessions, 6/week for 12 weeks. Each session was 60 min in duration and consisted of least 15 min of functional games, at least 15 min of exercises, and 5 min of stroke education using a Jeopardy style game.

There were 12 input devices used by patients to interact with the TR system: a PlayStation Eye camera, motion game controller (PlayStation Move, Sony; Tokyo, Japan), joystick, small buttons (10), large buttons (4), toy pistol holding a Wii remote (Nintendo; Kyoto, Japan) with corresponding IR sensor bar, trackpad (Logitech; Newark, CA), grip force cylinder, pinch force cube, rotating shuttle wheel (Powermate, Griffin Technology; Nashville, TN), steering wheel with gas/brake, and a 9-DOF IMU containing a 3-axis accelerometer, gyroscope, and magnetometer.

A total of 114 exercises were available, targeting UE, LE, and trunk. Each was 1–5 min long and consisted of a video showing the assigned movement. Patients were instructed to move as in the video. Therapists had the option to incorporate standard equipment (e.g., resistance bands; Theraband; Akron OH) provided to patients at the time the TR system was delivered to the home, to be used while watching the exercise videos.

A total of 33 functional games were also available, each 1–5 min long. These stress motor control features, e.g., varying movement speed, range of motion, target size, extent of visuomotor tracking, or level of cognitive demand. Game features were selected and adjusted by the therapist. For example, during the whack-a-mole game, higher difficulty level means a broader area where targets can appear on the tabletop and less time to successfully hit the target. Therapists also select which input device the patient will use for game play, based on UE status, e.g., the flappy-bird game can be played using the grip force cylinder, pinch force cube, or trackpad.

Therapists also decided whether five photographs would be taken at random time points during a given game, to gain insights into how the patient was playing the game. After the day's 1-h of assignments were completed, patients were allowed to free play, i.e., to use the system to play functional games *ad libitum*.

Stroke education targeted five categories (Stroke Risk Factors, Stroke Prevention, Effects of Stroke, Diet, and Exercise) focused on secondary prevention. Patients made arm movements to enter their answers to multiple-choice questions, delivered via a video Jeopardy game format [an approach known to foster learning (52, 53)], and then received feedback on their answers.

To build each day's treatment session, therapists used a graphical interface to drag treatment elements into a 60-min planner for each day's session; they then adjusted the challenge level (games) and the duration (games and exercises), and selected which input device would be used to drive gameplay (games). The daily treatment plan was regularly updated by a therapist based on findings from videoconferences and from review of TR-based data. Four types of TR-based patient data were automatically transmitted from home to lab, in real time: system usage (time TR was used), patient performance (game scores), behavioral status (assessment scores), and photographs (during games and pill consumption).

Patients had 18 HIPAA-compliant videoconferences (VSee software; VSee; Sunnyvale, CA) with a licensed therapist: three times/week during weeks 1–2, two times/week during weeks 3–4, and one time/week during weeks 5–12. During videoconferences, questions were answered, feedback was provided, progress was reviewed, and on some days remote assessments were made.

During the 30 min prior to the TR session, the computer alerted the subject that the start time was coming soon. The subject hit a large tabletop button to begin the day's session and to start subsequent games/exercises after each one is completed. In this way, patients could take a break between games/exercises. Unsupervised sessions had the same treatment content as supervised sessions, but no therapist contact.

### Novel TR Features Evaluated

Key novel features added to the TR system and evaluated included the following:

1. Lower extremity games and exercises: Our prior three TR studies (46–48) were focused exclusively on UE therapy. Here we also targeted the paretic LE, introducing LE exercise videos, LE driving games, and the AR “virtual varmint” game. In the driving games, patients used a steering wheel and gas/brake pedals to navigate a virtual terrain.
2. Augmented reality (AR) gaming: With an AR-based approach, subjects interact in the real-world workspace with virtual computer-generated objects (47, 54, 55). This was used in the Virtual Varmint game, where subjects looked at a tabletop monitor that showed a real-time video display of their paretic foot; a virtual gopher was projected into this display, and when the subject's foot overlapped with the gopher, points were earned. A camera was placed under the table and pointed at the paretic foot. The TR computer displayed camera output on the tabletop monitor along with a computer-generated varmint (a gopher). Patients looking at the tabletop monitor thus used real-time images of their foot movements to manipulate a virtual varmint.
3. Use of real objects to drive gameplay targeting Instrumental Activities of Daily Living (iADL): The TR accelerometer had a magnet and was attached to a lemonade pitcher by the patient prior to starting the game. Accelerometer data were sent to the TR computer. As the subject used the paretic arm to rotate the pitcher, a figure of a pitcher on the video screen moved synchronously, allowing the subject to use a real object to play a game where the goal was to fill empty cups to the correct level.
4. Daily study pill consumption: Each day, patients were also asked to consume a study pill. This pill was an unblinded placebo (small sugar-free mint). The computer screen guided patients through a series of steps to open the pill container, put the lid on the TR table, put a pill in their hand, ingest the pill, and then replace the lid; the TR camera took a picture when the patient hit a button to indicate that each step was completed, and these pictures were later used to confirmed compliance with pill intake. Pills were kept in a yellow container, clipped to the TR table, and had a lid (DoseSmart; RxCap; Boston, MA) that sent a Bluetooth signal to the computer each time the container was opened.
5. Expanded assessments, as below.
6. E-mail actionable reports for critical findings: The study coordinator and lead investigator were automatically sent an email (with a suggested response) if either of two conditions arose: (1) sharp increase in pain, defined as increase in the shoulder pain score by ≥20/100, with the suggested response being to contact the patient same day; (2) non-compliance with therapy, defined as the patient failing to initiate TR for 3 days in a row, with the suggested response being to contact the patient same day.
7. Reliance on home WiFi: In addition, we sought to evaluate the performance of each patient's home WiFi network. In each case, the home-based TR system was connected to the internet using the patient's personal wireless network rather than a study-provided wireless cellular modem.

### Study Assessments

To fully characterize enrollees, a broad range of assessments was evaluated, including measures of impairment, activities limitation, quality of life, and patient-reported measures. In addition to assessments at the four in-clinic visits, patients underwent assessments at home via the TR system, some of which were supervised by therapists and some scored with no therapist present.

The primary endpoint was the UE-FM scale (56, 57), which ranges from 0 to 66, with higher scores indicating less UE impairment. The main secondary endpoint for UE was the Box & Blocks (B&B) score (58), which counts the number of blocks a subject can lift and move across the table in 60 s. The two main LE secondary endpoints 10 meter walk test of gait velocity (59) (measured as the mean of two trials) and the LE-FM motor scale (56, 57), which ranges from 0 to 34, which higher scores indicating less LE impairment. Demographic data, medical history, and handedness (60) were obtained on study entry. The presence of aphasia was assessed using Philadelphia Naming Test (Form A) (61). The presence of neglect was assessed using the Line Cancellation Test (62).

A social network survey (PERSNET) was assessed during the live visit 6 weeks after enrollment. The results of these social network studies are presented in a separate companion paper (63).

Several additional dimensions of stroke outcome were measured at baseline and after 12 weeks of therapy: Optimization in Primary and Secondary Control (OPS) scale (64), which measures dedication to treatment goals across 12 questions, with scores ranging from 1 to 7 and higher scores reflecting greater motivation; Nottingham sensory scale (65), which assesses a range of sensory modalities in the distal UE, with maximum score of 11 and higher scores reflecting better sensory function; Geriatric Depression Scale (GDS) (66), which measures depression across 15 questions, with a maximum score of 15 and higher scores reflecting greater depression; Montreal Cognitive Assessment (MoCA) (67), which measures cognitive function, with maximum score of 30 and higher scores reflecting less cognitive impairment; modified Rankin Scale (mRS) (68), which measures global function (disability and dependence), with a maximum score of 6 and higher scores reflecting poorer function; EuroQol visual analog scale (EQ-VAS) (69), in which a subject rates his/her own health from 0 to 100 and higher scores reflect better quality of life; and modified Ashworth Spasticity (mAS) scale (70), which measures spasticity at the elbow flexor, with a maximum score of 4 and higher scores reflecting greater spasticity.

Some assessments were made by the study therapist using the TR system. Patient-reported outcomes, which are well aligned with scoring via videoconference, were examined. Hand function was measured using the Stroke Impact Scale (71) (SIS)-hand subsection, with a maximum score of 5 and higher scores reflecting better hand usage. This patient-reported outcome was measured during videoconferences in weeks 1 and in 12. Functional status was measured using the SIS-activity of daily living (ADL) subsection (71) during videoconferences in weeks 2 and in 12; the maximum score is 5, and higher scores reflect less difficulty with ADLs.

Other assessments were made by the patient, with no therapist present, using the TR system. To maximize the likelihood that the unsupervised patient at home would be successfully assessed, the focus here was on Likert scales and visual analog scales. The MOS Social Support Survey (72) (MOS-SSS) was scored via the TR system during week 2; scores range from 19 to 95, with higher scores reflecting stronger social support. The Brief Resilience Scale (73) was also scored via the TR system during week 4; maximum score is 30, with higher scores indicating better resilience. The Generalized Anxiety Disorder-7 (74) (GAD-7) scale was also scored via the TR system in week 3; maximum score is 21, with higher scores reflecting greater anxiety. Finally, shoulder pain and fatigue were assessed weekly with a focus on the first 6 weeks, using a visual analog scale (0–100) where higher numbers indicate greater pain and fatigue, respectively.

Some assessments were scored by the therapist at both a live visit and during a TR videoconference, in order to validate telehealth screening. Measures of mood and language were selected given the expectation that patients would likely be stable in these domains across the 1–3 weeks when serial testing was performed. The GDS score was scored during a week 9 videoconference and a week 12 in-clinic visit. The Philadelphia Naming Test (61) short form was scored twice; the maximum score is 30, and higher scores reflect less aphasia. Form A was scored during the live week 6 visit; Form B, which assesses 30 different objects, was scored by the therapist 1 week later, during a videoconference.

### Data Analysis

Data analysis used non-parametric statistical testing (JMP 13, SAS; Cary, NC). Statistical moments are presented as median (IQR). All analyses were two-tailed, with statistical significance set at *p* < 0.05 and no corrections made for multiple comparisons in this feasibility study. Within-subject changes in performance over time were analyzed using the Wilcoxon Signed Rank Test. Comparisons of subject values in weeks 1–6 vs. weeks 7–12 were analyzed using the Wilcoxon Rank Sums Test. Comparisons of two continuous variable were performed using the Spearman Rank Correlation Coefficient. For some telerehab-based assessments, data were missing for 1 subject; missing data were not imputed.

## Results

### Subjects

A total of 15 subjects were screened, of whom 13 were enrolled. Each was assigned 72 treatment sessions and 18 videoconferences with a study therapist, distributed over 12 weeks. For all subjects, the home WiFi network consistently supported TR data uploads and downloads as well as videoconferences. Of the 13 patients, nine received concomitant therapy outside of study procedures at some point during the study: seven at baseline; seven at 6-weeks, and eight at 12 weeks. There were no adverse events.

Subjects were a median of 61 years old and 4 months post-stroke at study entry (Table 2); 5 patients were <90 days post-stroke (range, 37–67 days), 8 patients were ≥90 days (range, 119–1,682), and 4 patients were >1 year post-stroke (range 16–56 months). Nine subjects were White, 3 Asian, and 1 African-American. One subject was Hispanic. All subjects had completed high school, with a median of 2 additional years of education. No patient had aphasia or spatial neglect.

**Table 2:** Patient values at baseline.

	**Baseline**
N	13
Sex	9 M/4 F
Age	61 [52–65.5]
Time post-stroke (days)	129 [52–486]
BMI	26.6 [24.8–32.3]
Systolic blood pressure (mm Hg)	120 [118–134]
Diastolic blood pressure (mm Hg)	75 [72–80]
Hypertension	10 Yes
Hypercholesterolemia	7 Yes
Diabetes mellitus	4 Yes
Atrial fibrillation	2 Yes
Affected side	10 L/3 R
Shoulder pain present at baseline	8 Yes
Handedness	10 R/3 L
Optimization in Primary and Secondary Control scale	5.6 [5.1–6.0]
MOS Social Support Survey^*^	83 [69–92]
Brief Resilience Scale^*^	23.5 [22.25–26]
Generalized Anxiety Disorder-7^*^	3 [0–8.5]

Values are median (IQR).

* Data acquired via the TR system, with no therapist present.

### Therapy Dose and Compliance

Patients completed 50.4 h (33.3–56.7) of TR over the 12 weeks, and attended a median of 16 (14–18) videoconferences. Patients initiated the daily TR session (did >5% of assigned minutes) on 79.9% of days, and completed most of the session (did >50% of assigned minutes) on 65.7% of days. Common reasons for missed therapy sessions were vacation (55), demands from the patient's job (40), scheduling conflicts (32), and illness (22).

**Figure 1:**
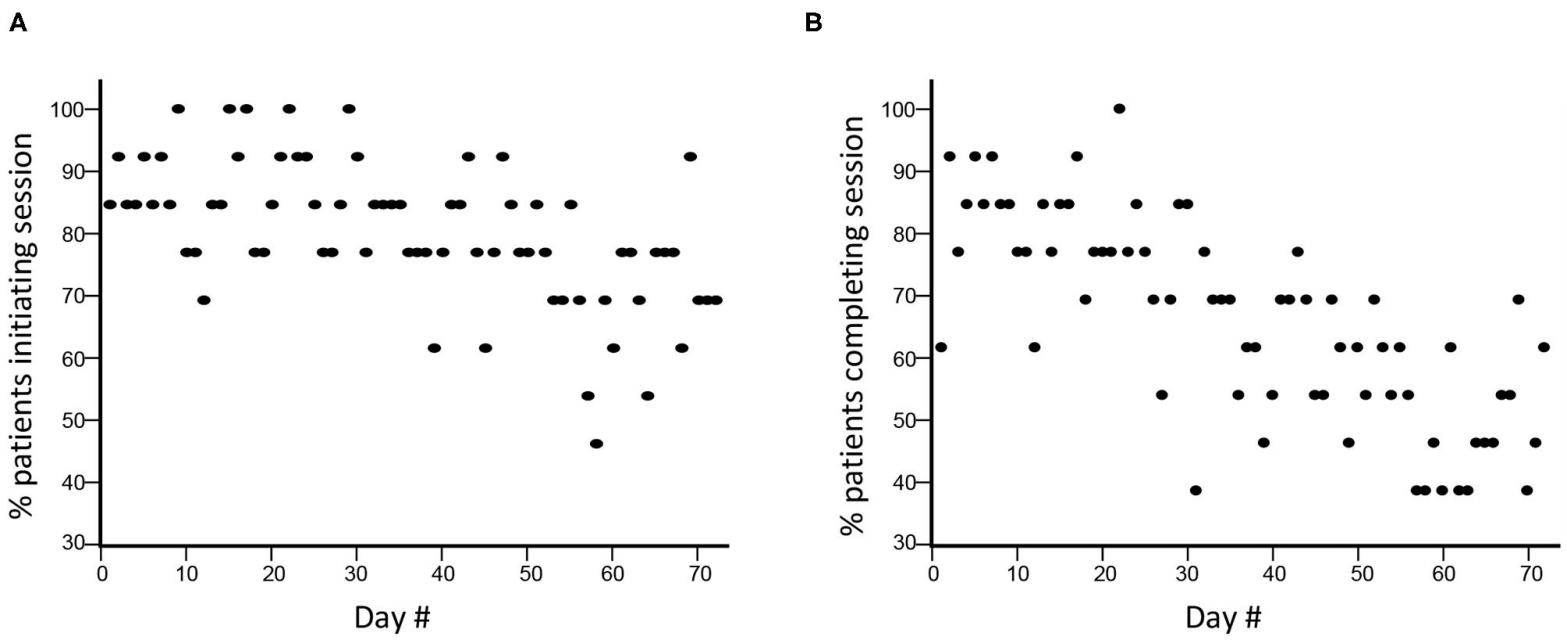
Compliance with TR assignments declined during the 12 weeks. **(A)** During TR weeks 1–6 (days 1–36), there was a decline in TR session initiation compared to weeks 7–12 (days 37–72), from 86.4 to 73.5% (*p* < 0.0001). **(B)** There was a similar decline in in TR session completion, from 76.9 to 54.6% (*p* < 0.0001).

Compliance declined across the 12 weeks of therapy. Comparing weeks 1–6 with weeks 7–12: session initiation decreased from 86.4 to 73.5% (*p* < 0.0001; Figure 1A); and session completion decreased from 76.9 to 54.6% (*p* < 0.0001; Figure 1B), i.e., non-compliance doubled in the second 6-week block. The rate of session completion across the 12 weeks did not vary in relation to time post-stroke (*r* = 0.28, *p* = 0.36), age (*r* = 0.33, *p* = 0.27), or baseline scores on the GDS (*r* = −0.38, *p* = 0.2), MoCA (*r* = −0.28, *p* = 0.35), or UE-FM (*r* = −0.41, *p* = 0.17). Although the content and quantity of assigned therapy was constant over time, subjects completed a median of 61.5 (IQR = 33–65.8) min/day of therapy during weeks 1–6 vs. 43.6 (2–63.3) min/day during weeks 7–12 (*p* < 0.0001). Subjects engaged in free play after finishing their assigned therapy on 1/5 days during weeks 1–6 but only 1/16 days during weeks 7–12 (*p* < 0.0001).

### In-Clinic Assessments

TR was associated with significant UE motor gains. From baseline pre-therapy to follow-up after 12 weeks of TR therapy, the primary endpoint, UE-FM score, changed by 6 (2.5–12.5) points (*p* = 0.0005). Most of this change was achieved in the first 6 weeks, as UE-FM score change from baseline to week 6 was 6 (2–9.5) points (*p* = 0.0007). From week 6 to week 12, median change was 1 (−0.5 to 2) point (*p* = 0.19). The extent of 12-week gains on the UE-FM scale declined with increasing time post-stroke at enrollment (*r* = −0.63, *p* = 0.02). Similar gains were seen from baseline to week 12 for change in affected arm Box & Blocks score, with median change of 9 blocks (3.5–17.5) (*p* = 0.0005). Median change in unaffected arm Box & Blocks score during this interval was non-significant [2 blocks (−5 to 4.5) (*p* = 0.69)].

Findings were similar for the LE. Gait velocity improved by a median of 0.15 (0.07–0.22) m/s from baseline to week 12 (*p* = 0.0007). Most of this change was achieved in the first 6 weeks, where change from baseline was .08 (0.02–0.20) m/s (*p* = 0.007). Results were more modest for the LE-FM score change, which was 1 (−0.5 to 5) point (*p* = 0.065) over 12 weeks.

Several other classes of outcome measure also showed improvement. Scores on the mRS ranged from 2 to 3 at baseline and from 1 to 2 at week 12 (change over time, *p* = 0.03). This change was accounted for by improved mRS score in 6/13 patients (five with initial score = 3 and one with initial score = 2), 3 of whom were <90 days post-stroke (37–67 days) and 3 of whom were >90 days post-stroke (4, 5.5, and 56 months post-stroke) at study enrollment. In addition, the EQ-VAS increased from baseline to week 12 by a median of 15 (2.5–31), indicating improved self-rating of health state. Mood (GDS) improved over time (*p* = 0.05); note that a GDS score > 5, suggesting depression, was present in 3 subjects at week 1 and 0 subjects at week 12.

### Remote Assessments

There were three types of remote assessments (Table 3). First, therapist-directed measures during videoconferences captured behavioral gains. Hand usage, measured using the SIS-hand scale, showed significant gains (*p* = 0.002). Functional status, measured using the SIS-ADL scale, showed improvement over time that narrowly missed significance (*p* = 0.06). Second, therapist-independent measures (no therapist online when scored) reliably assessed patient status. Median score on the MOS-SSS was 83 (69–92), indicating strong social support on average. Median score on the Brief Resilience Scale was 23.5 (22.25–26), indicating overall good resilience. Median score on the GAD-7 scale was 3 (0–8.5), indicating low anxiety on average. Scores for shoulder pain were stable from week 1 to week 6, rising slightly, from 0 (0–31) to 9 (0–25) (*p* = 0.46) on this 100-point visual analog scale. Fatigue, however, declined significantly from week 1 to week 6, from 36 (8–61) to 16 (0–43) (*p* = 0.004). Third, for two assessments, therapist scores obtained in-clinic were compared to those obtained during TR. The two sets of GDS scores obtained 3 weeks apart were closely related (*r* = 0.89, *p* < 0.0001; **Figure 5**) and showed an intraclass correlation coefficient of 0.66; note that one subject was not available for the week-9 videoconference. Findings on the Philadelphia Naming Test had a ceiling effect that limited comparisons, as scores on Form A in-clinic were perfect in all but two patients and scores on Form B via TR were perfect in all but three patients.

**Table 3:** Behavioral change after 6 and 12 weeks of treatment.

	**Baseline**	**After 6 weeks of treatment**	***p*^**∧**^**	**After 12 weeks of treatment**	***p*^**∧∧**^**
Arm motor Fugl-Meyer score	46 [42–57]	57 [50–61]	0.0007	59 [52.5–61.5]	0.0005
Box & Blocks score, affected arm	32 [23–42.5]			46 [39–50]	0.0005
Box & Blocks score, unaffected arm	55 [48.5–57]			53 [47.5–61.5]	0.69
Gait velocity [m/sec]	0.94 [0.67–1.09]	0.90 [0.71–1.2]	0.007	1.01 [0.83–1.21]	0.0007
Leg motor Fugl-Meyer score	28 [23.5–29]	27 [26–29.5]	0.48	28 [27–30.5]	0.065
Nottingham sensory score, affected arm	11 [10–11]			11 [11–11]	0.12
Geriatric Depression Scale score	3 [1–5]			1 [0–4]	0.05
Montreal Cognitive Assessment score	27 [24.5–29]			29 [25.5–30]	0.13
Modified Rankin Scale	2 [2–3]			2 [2–2]	0.03
EQ-VAS	75 [52.5–80]			80 [72.5–90]	0.003
Modified Ashworth spasticity scale, elbow flexor	1.5 [0.5–1.5]			1 [0–1.25]	0.11
Stroke Impact Scale-hand^*^	3.4 [2.7–3.9]			4.0 [3.5–4.8]	0.002
Stroke Impact Scale-ADL^*^	3.8 [3.1–4.4]			4.2 [4.0–4.5]	0.06

Values are median (IQR); p values are based on Wilcoxon Signed Rank Test for change over time from baseline to ^∧^6 weeks or baseline to ^∧∧^12 weeks.

* Data acquired via the TR system, with therapist supervision.

### Augmented Reality Gaming and Application of Sensors to Real Objects

**Figure 2:**
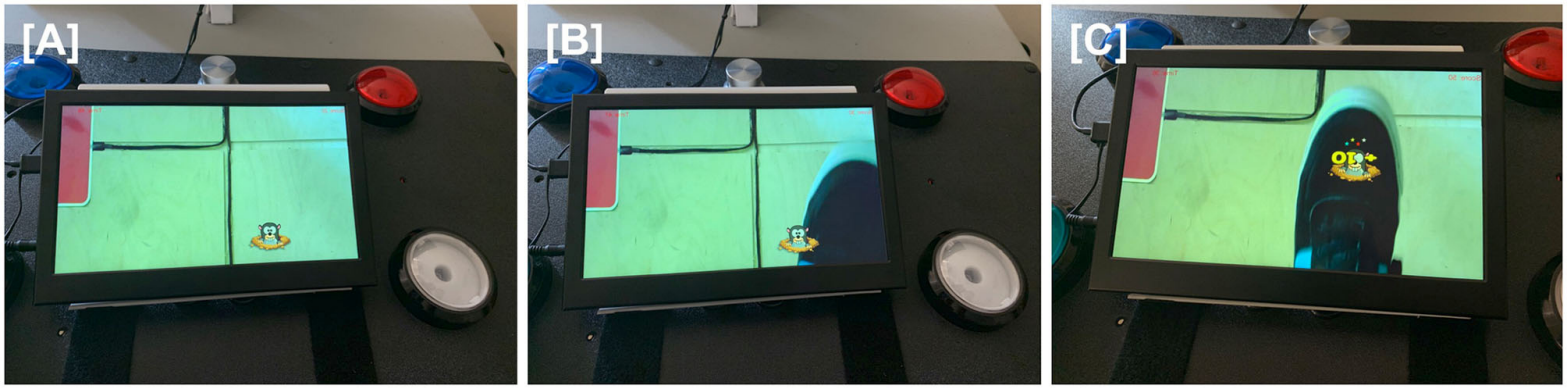
For the virtual varmint game, a camera under the table pointed at the floor captured live images, including the patient's paretic foot, that were projected onto the screen of a tabletop tablet. When patients directed their gaze at the tabletop, they were thus able to see real-time images of their foot movements. A virtual varmint was introduced into the tablet image, which the patient was able to manipulate with their foot. **(A)** A virtual image of a varmint is introduced onto the screen of the tabletop tablet. **(B)** The patient moves his foot toward the virtual varmint. **(C)** The patient swats the virtual varmint with his foot, scoring points.

**Figure 3:**
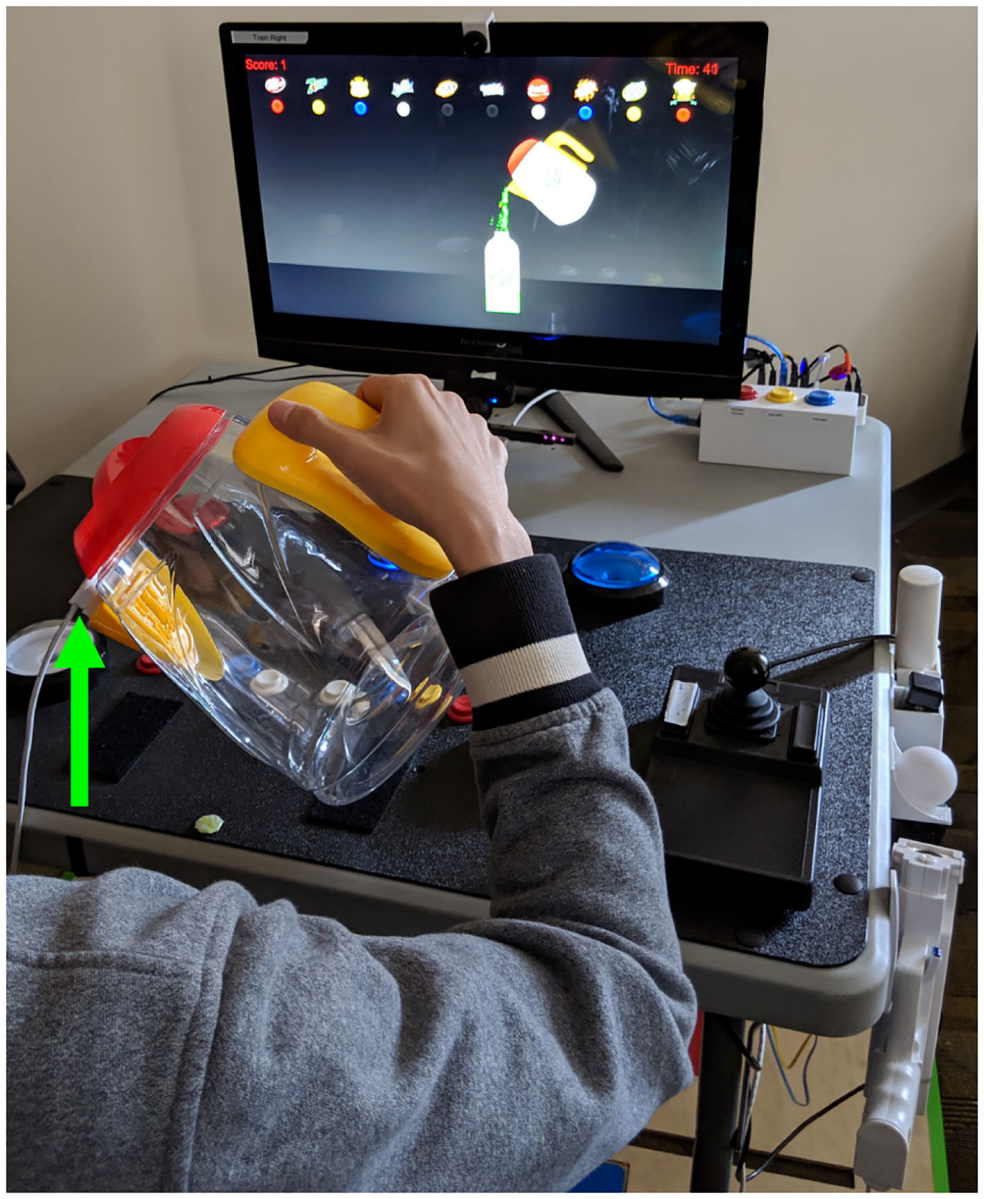
Sensors were attached to real objects to play a game that drives an iADL. The patient grasped an actual water pitcher onto which an accelerometer (green arrow) was attached magnetically. Accelerometer data were sent to the computer running the TR program. As the subject rotated the hand-held pitcher, the figure of a pitcher on the video screen moved synchronously. In this way, the subject used a real object to play a game, displayed on the TR computer screen, where the goal was to fill empty cups to the correct level.

Augmented reality was successfully incorporated into home-based TR. The equipment was installed in the home, the AR game assigned by the therapist, and these were used by patients during TR (Figure 2). Similarly, an accelerometer could be applied to various objects by the patient at home (Figure 3), allowing movement of a real object in a functional way to play a game that emphasized iADLs.

### Daily Study Pill

**Figure 4:**
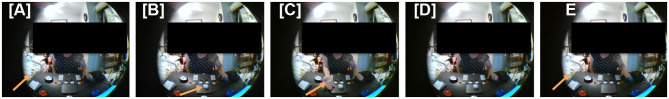
Photographs were used to confirm that the study pill was taken each day as instructed. **(A)** The patient is seated; arrow indicates cap on the bottle holding study pills. **(B)** The patient has removed the pill bottle lid and placed onto the trackpad; arrow indicates the lid. **(C)** The patient has removed one pill and placed into her palm; arrow indicates the pill. **(D)** The patient has taken the pill. **(E)** The lid has been replaced; arrow indicates the lid.

Consumption of a study pill once/day (Figure 4) was successfully incorporated into TR sessions. One subject requested not to have any photos taken. The remaining 12 subjects used their TR system on 681 days, and took their pill on 619 of these days, resulting in 90.9% compliance with daily study pill consumption. The Bluetooth-enabled pill bottle cap worked properly but there were difficulties keeping its software running at all times in the background of the TR program.

### Automatic Actionable Reports by Email for Critical Findings

Email-alerts were sent to the PI and to the study coordinator reliably and with specificity whenever there was (1) a substantial increase in body or shoulder pain or (2) non-compliance with therapy for 3 days. During the study, reportable incidents only occurred in relation to non-compliance.

## Discussion

High dose rehabilitation therapy can improve outcomes after stroke, but this is not provided to many patients. Telehealth methods have the potential to overcome many of the barriers to high dose therapy, such as transportation limitations or limited regional access. In an effort to improve our approach to home-based TR, the current study aimed to evaluate the feasibility of several system expansions related to assessment and to treatment.

Enrollees were recruited at wide-ranging times post-stroke, had overall moderate motor deficits and little sensory deficits, were highly dedicated to treatment goals, lacked cognitive deficits, had low anxiety and depression symptoms, had good social support, and reported high resiliency (Tables 2, 3). In our prior studies, we relied on a Verizon wireless modem to connect the patient's home-based TR system to the internet and thereby enable communication between the home and the clinic. The current study found that we can instead rely on the patient's personal wireless network, an approach that, when available, has advantages such as connection speed.

Across the 12 weeks of TR, UE and LE motor status, functional status, and quality of life all improved significantly (Table 3), particularly during the first 6 weeks. These gains occurred as patients completed a median of 50.4 h/subject of TR. Some of this improvement might be related to spontaneous post-stroke recovery. Five of the 13 enrollees were <90 days post-stroke at enrollment, and so some of their behavioral improvement is likely attributable to spontaneous recovery; consistent with this, UE-FM gains declined with greater time post-stroke.

### Longer Therapy Duration

Our prior trial (48) evaluated 6 weeks of therapy provided 6 days/week (70-min sessions, 42 h total). Here we aimed to evaluate a course of TR lasting for twice as long: 12 weeks of therapy (60-min sessions, 72 h total). This was driven in part by our review of home-based technologies for stroke rehabilitation (75), which noted that across 31 studies, most technologies were evaluated for short time periods. In addition, larger doses of TR have been reported to result in greater benefit (76).

The rate with which subjects initiated (Figure 1A) and completed (Figure 1B) TR assignments declined significantly across the 12 weeks of therapy. During TR weeks 1–6, TR session completion was 76.9%, lower than the 98.3% value seen during a 6-week course of telerehabilitation in our 11-site study (48). Compliance was not related to time post-stroke, age, depression, cognitive status, or arm motor impairments at baseline, although the sample size is limited for examining these issues. Several reasons might account for lower compliance over time seen in the current study. Functional gains during the first 6 weeks might have reduced motivation to perform TR thereafter. Patients might have become bored with some games. During weeks 7–12, videoconferences were reduced from 3x/week to 1x/week, due to budgetary constraints, which might have contributed to the doubling of non-compliance during this period. These videoconferences were a stimulus for patient accountability, and so a reduction in their frequency might have adversely affected compliance. In addition to driving accountability, videoconferences also foster a relationship between patient and therapist that might be important to sustained compliance. In a qualitative study (77) of 13 patients randomized to TR at one site in our national trial, regular videoconferences with a therapist were highly rated. Fewer interactions during videoconferences might produce weaker patient-therapist bonds, contributing to non-compliance.

### Treatment of Both UE and LE Motor Deficits

This study also examined the feasibility of treating both UE and LE motor deficits. While not all stroke survivors have motor deficits in both UE and LE, involvement of both is more common than is paresis in either alone (78). Despite this, only 2 of the 22 stroke TR trials have targeted both UE and LE deficits (45). The current study found that exercises and games targeting LE motor deficits were readily incorporated alongside those targeting UE, and were associated with significant gains in gait velocity.

### Increasing the Functional Relevance of TR

Practice of real life tasks with real objects can increase object affordance and task ecology and is often incorporated into constraint induced therapy (51). The TR system is well suited to adopt this strategy. The current study found that a sensor could be attached to real objects, providing data that are used to drive game play that targeted pouring liquids (Figure 3), which is part of meal preparation, an important iADL.

An additional way to expand the functional relevance of TR therapy is to incorporate virtual objects that may be impractical or unsafe in the patient's home. AR integrates virtual elements into the real world (54) and was successfully incorporated into the TR system (Figure 2). AR introduces an additional form of human-computer interface that can be used to modulate a task's cognitive demand (55).

### Daily Consumption of a Study Medication Integrated Into TR

Daily ingestion of a study pill was integrated into the home-based TR system. Patients were prompted to take a study pill at the start of each session and did so 90.9% of the days that they initiated a TR session (Figure 4). Driving patient compliance with pill consumption each day might be useful in clinical practice, e.g., to improve secondary stroke prevention or in clinical research, e.g., when studying an orally ingested drug that might promote recovery, particularly since TR enables careful pairing of behavioral training with pill consumption (79–81).

### Additional TR-Based Assessments

**Figure 5:**
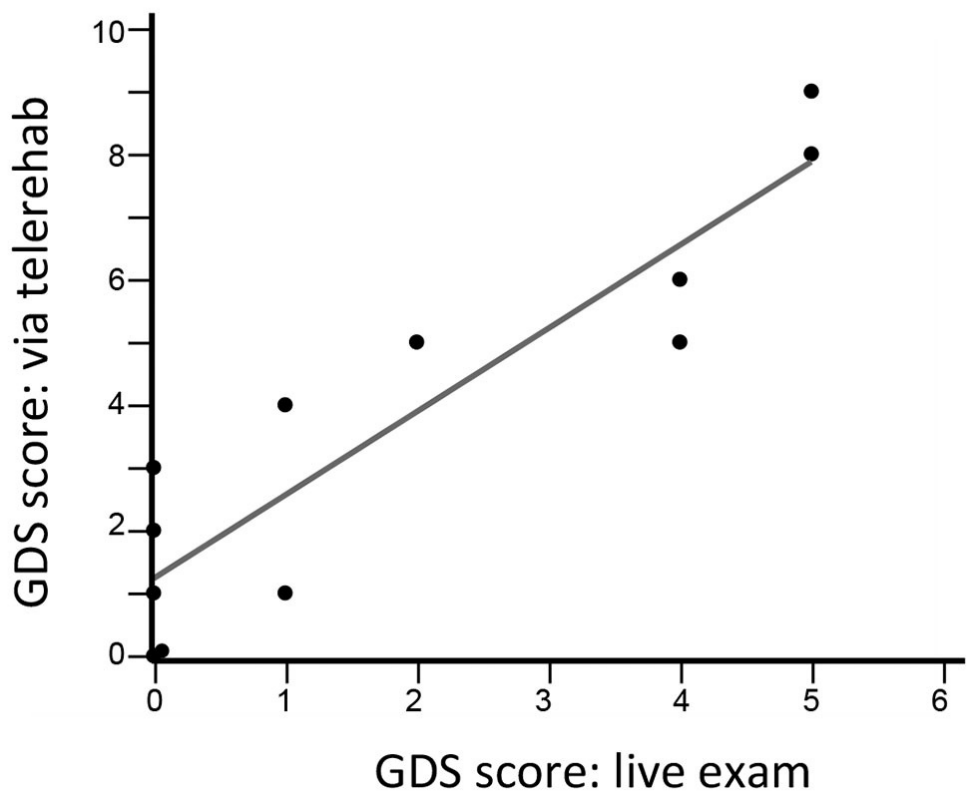
GDS scores during a live visit 12 weeks after study entry are closely related to GDS scores obtained during a TR videoconference 9 weeks after study entry (*r* = 0.89, *p* < 0.0001, *n* = 12). The intraclass correlation coefficient was 0.66.

TR not only provides an opportunity for remote therapy but also provides a platform for remotely measuring, both passively and actively (82), a broad range of human activities (83) and behavioral and psychological symptoms (84). This can promote greater independence and quicker access to healthcare professionals (85). The current results support the feasibility of using TR to measure hand usage (SIS-hand) and functional status (SIS-ADL).The GDS was validated for depression telescreening (Figure 5); interestingly, average scores at home were higher compared to when the same scale was administered in the clinic, in contradistinction to prior results obtained in a non-stroke population (86).

We evaluated four assessments that were scored asynchronously, i.e., by the patient with no therapist supervision, and all were successfully collected. These include the MOS-SSS, which indicated strong social support; the Brief Resilience Scale, which showed overall good resilience; and the GAD-7, which showed low anxiety scores. In addition, shoulder pain and fatigue were assessed weekly. Shoulder pain, the most common adverse event in patients randomized to TR in our prior national trial (48), was mild and stable over 6 weeks. Patients reported a significant decline in fatigue over time.

### TR-Generated Actionable Reports

A very large amount of data is generated by the TR system. Efficient approaches are needed to bring the most critically important findings to the attention of busy clinicians. We incorporated actionable reports, whereby a clinician is notified electronically of a critical finding, along with a suggested response. Electronic notification of critical results has advantages that include decreased workflow interruptions and more timely closed-loop communications of key patient data (87). Such reports are most effective when recommendations presented to clinicians are clear, explicit, and actionable (88). Such reports should focus on high quality observations that present critical new knowledge (89). Communication of critical results is a national patient safety goal emphasized by the Joint Commission, and is no less important in stroke recovery. The current pilot study provides support for actionable results to transmit critical findings in two categories, pain and treatment compliance.

### Strengths and Weaknesses

Strengths of this feasibility study include successful evaluation of several new expanded TR features related to treatment and to assessment, including longer-term therapy, addition of therapy targeting the LE, increased dimensions of assessments, incorporation of real objects and AR, and introduction of a daily study pill. There were several key weaknesses, as well. The sample size was limited. As this was a feasibility study, there was a single treatment arm and no control group. Some patients might not have completed spontaneous recovery at study entry, although the goal was to evaluate new TR features rather than establish efficacy. The total number of daily limb movements during TR was not measured, as in our prior studies. No qualitative study was performed to better understand the perspectives of patients and caregivers. Current results incompletely generalize, as enrollees lacked substantial aphasia, neglect, sensory deficits, depression, and anxiety.

### Conclusions

The current study examined the feasibility of adding new modules to a home-based TR system for patients with stroke. Some modules were therapy-focused, such as longer duration of therapy and ingestion of daily study medication, while others were diagnostic, such as assessments performed by the patient with no therapist supervision. These results inform future efforts to develop TR approaches to address the many aspects of treating patients with stroke.

## Data Availability Statement

The raw data supporting the conclusions of this article will be made available by the authors, without undue reservation.

## Ethics Statement

The studies involving human participants were reviewed and approved by UC Irvine IRB. The patients/participants provided their written informed consent to participate in this study.

## Author Contributions

The study was designed by SC, LD, AM, JS, RA, RZ, AD, WS, and JH. The study was conducted, the manuscript was written, and critically revised by all authors.

## Conflict of Interest

SC has served as a consultant for Constant Therapeutics, MicroTransponder, Neurolutions, SanBio, Stemedica, Fujifilm Toyama Chemical Co., NeuExcell, Medtronic, and TRCare. AD is an expert witness for Neuro Consults, LLC. LD, VL, JS, and RA are consultants for TRCare. The remaining authors declare that the research was conducted in the absence of any commercial or financial relationships that could be construed as a potential conflict of interest.
